# High-frequency hydrodynamic and hydrochemical data from karst unsaturated zone

**DOI:** 10.1016/j.dib.2025.111812

**Published:** 2025-06-21

**Authors:** Leïla Serène, Guillaume Cinkus, Naomi Mazzilli, Jean-Baptiste Decitre, Christophe Emblanch, Franck Tison, Julien Dupont, Milanka Babic, Roland Simler, Matthieu Blanc

**Affiliations:** a UMR 1114 EMMAH (AU-INRAE), Université d’Avignon, Avignon 84000, France; b Low Background Noise Underground Research Laboratory, CNRS and Avignon University, Rustrel, France; c Independent Researcher, France

**Keywords:** Groundwater, Critical zone, Weather station, Discharge, Electrical conductivity, Rainfall, Temperature, Air pressure

## Abstract

This dataset provides hydrological continuous data for 12 unsaturated zone flows of the Fontaine the Vaucluse karst system (France). These flows are caused by faults, fractures, karst conduits, and slow-flows from porous rock. This panel of unsaturated zone flow is unique. The dataset includes electrical conductivity, temperature and discharge, obtained through the artificial galleries of the Low Noise Underground Laboratory (LSBB, https://lsbb.cnrs.fr), and providing a unique window into the hydrological processes occurring in the unsaturated zone of karst aquifers. It also provides air pressure, temperature and humidity inside the galleries and weather parameters at the surface above the galleries (pluviometry, temperature, solar radiation, humidity, wind speed, wind direction, barometric pressure). The data are provided at an hourly time step from July 2022 to April 2024 and correspond to filtered data.

Discharge is calculated from a homemade flowmeter consisting of a Plexiglas tube that collects the water and has a pressure sensor at the bottom. The pressure is converted into a volume of water thanks to a linear model, and then converted into discharge using the extreme values corresponding to the filling and emptying of the Plexiglas tube. All data have been filtered by removing erroneous values such as extremes due to probe malfunction or operator intervention, by checking the accuracy of the continuous measurement with point-in-time monitoring, and by removing values when the flow is inactive.

This dataset is original in that it monitors various types of unsaturated zone flow, which are rarely monitored due to a lack of speleological access, especially slow flows within the limestone matrix. These data can be used to assess pressure transfer thanks to pluviometry, flow temperature and discharge; as well as mass transfer thanks to pluviometry and electrical conductivity. Weather data can also be used to improve climate models as the nearest weather station is 10km away.

Specifications Table

**Table:** 

Subject	Environmental Science
Specific subject area	Hydrodynamic and hydrochemistry data in hydrogeology, weather data in climatology, karst hydrogeology, karst spring, unsaturated zone
Type of data	Table, filtered data
Data collection	The Low Noise Underground Laboratory (LSBB) has 3 galleries allowing the monitoring of Fontaine de Vaucluse karst’s unsaturated zone flows (electrical conductivity, temperature (C4E probe, Ponsel), discharge with homemade flowmeter (plexiglass tube and PFT pressure sensor, Sick)). Air pressure, temperature and humidity inside the galleries is monitored (Yocto-meteo sensor, Yoctopuce). Surface weather (pluviometry, temperature, solar radiation, humidity, wind speed, wind direction, and barometric pressure) is monitored by a weather station (MetPak Pro, integrated Windsonic, Gill Instruments). Hourly data (Jul 2022-Apr 2024) was checked using manual measurements and outliers removed.
Data source location	Where the data are collected: • Institution : Laboratoire LSBB UAR3538 CNRS • City/Town/Region: Rustrel, 84400 • Region: Provence-Alpes-Côtes d’Azur • Country: France Geographical coordinated of the LSBB’s galleries entrance:Latitude : 43°55′43.11"NLongitude : 5°29′14.36"E
Data accessibility	Repository name: OSURISData identification number & direct URL to data: • Groundwater data from LSBB’s flows [1]: https://doi.org/10.26169/hplus.LSBB_water_high-frequency_monitoring • Data from the weather on the surface above the galleries and from the air inside the galleries [2]: https://doi.org/10.26169/hplus.LSBB_air_weather_high-frequency_monitoring Instructions for accessing these data:Data can be downloaded from the "Download Links" box at the bottom of each web page accessible by clicking on the links above.

## 1 Value of the Data

• Karst unsaturated zone flows are usually instrumented via speleological access (e.g. caves), which constrains both the types and number of monitored points. Speleological access is a major difficulty for sampling in unsaturated zones, as it involves longer access times and higher costs due to the difficulties of access, often requiring the presence of specialized technical personnel (speleologists). It also involves a limited exploration extent. Here, underground access is provided by a 4-long artificial gallery (the LSBB), which allows the collection of data from a wide range of flow conditions, illustrating the exceptional diversity and complexity of unsaturated zone flows. No other data articles on unsaturated zone flow could be found, which highlights the uniqueness of these data.
• Monitored parameters allow the assessment of both pressure (thanks to pluviometry and flow temperature and discharge) and mass transfers (thanks to pluviometry and electrical conductivity) within the unsaturated zone. This can be useful for improving understanding of unsaturated zone flows, thanks to modeling, time series analysis or by developing a typology or classification of these kinds of flows that could be transferred to other karst systems having less accessible unsaturated zones.Atmospheric parameters can be used to improve the robustness of climate models by having an additional point, as the nearest weather station is 10 km away. These data can also be useful for long-term climate trend analysis, as this station is designed to be permanent.
• The LSBB host numerous scientific activities from a wide range of research fields such as astrophysics and geosciences. Temporal and spatial variations in water content in the massif constitute part of the noise in the observations made and must therefore be characterized, which is what our dataset is contributing to.

## 2 Background

**Fig. 1:**
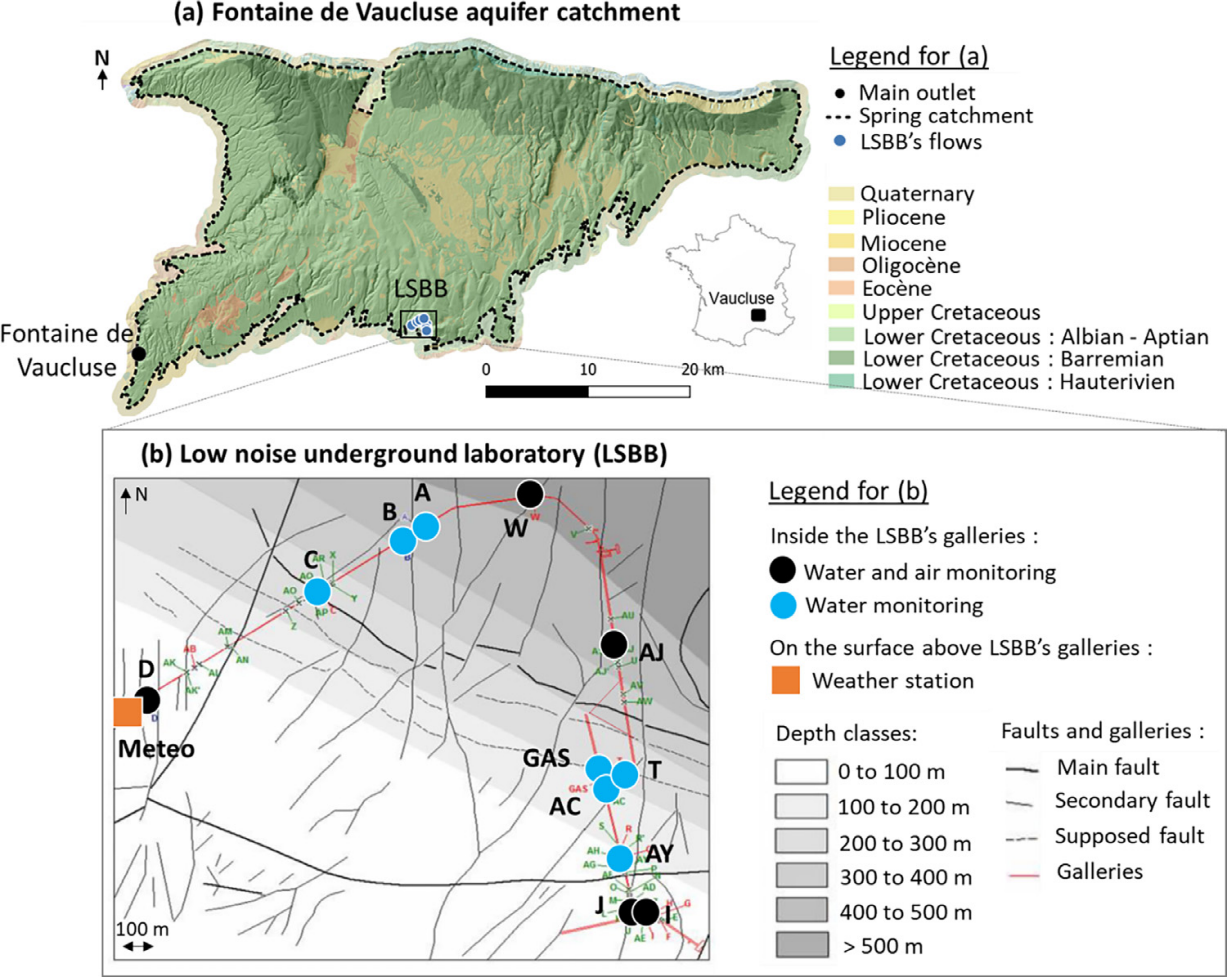
(a) Fontaine de Vaucluse karst system catchment, major outlet and LSBB location on background of 1:50000 geological map (BD-CHARM from BRGM). Catchment delineation of Fontaine de Vaucluse was taken from [7]; (b) Position of the measuring stations on an enlarged view of the LSBB on background of a structural map (modified from [8]).

This dataset was generated to study the behavior of unsaturated zone flows in karst areas in order to improve the knowledge of karst aquifers recharge. The Vaucluse karst system (southeastern France) was chosen because of its particularly thick unsaturated zone (around 800 m) which helps to maintain an important discharge of its main outlet, even during dry periods [3]. In the south of the catchment, the unsaturated zone is randomly intersected by the artificial galleries of the LSBB (https://lsbb.cnrs.fr, Fig. 1), which allow the observation of three to more than fifty unsaturated zone flows depending on the hydrodynamic conditions. These flows are located at depth below surface ranging from ∼30 m to almost 500 m. Since 2002, these flows are point-in-time monitored (major elements, stable isotopes, TOC, physicochemical parameters, discharge) at a weekly to monthly time step. This monitoring frequency is too large to fully assess the behavior of these unsaturated zone flows. For this reason, we instrumented with continuous monitoring stations for temperature, electrical conductivity and discharge the 12 most frequently activated flows. To compare water outputs with inputs, data can be used from the weather station installed on the surface above the LSBB’s galleries, in order to measure pluviometry.

## 3 Data Description

### 3.1 Location of the Measuring Stations

Study area is located in the Vaucluse karst system in southeastern France, inside and above the artificial galleries of the LSBB (https://lsbb.cnrs.fr, Fig. 1). The Vaucluse karst system is part of the French National Observatory of Karst (SNO Karst, https://sokarst.org/) and is characterized by a thick unsaturated zone of around 800 m [4]. LSBB galleries are dug in the unsaturated zone of the regional size aquifer, providing access to between 3 and more than 50 simultaneous flows. These flows are caused by faults, fractures and karst conduits, and slow-flows from porous rock. This panel of flow provides a unique window into the hydrological processes occurring in the unsaturated zone of karst aquifers. The 12 most frequently observed flows inside these galleries are instrumented and a weather station is installed at the surface, above flow D (Fig. 1b). In parallel, point-in-time measurement and water sampling are performed at least once a month from 2002 until now [5,6].

### 3.2 Available Data

**Fig. 2:**
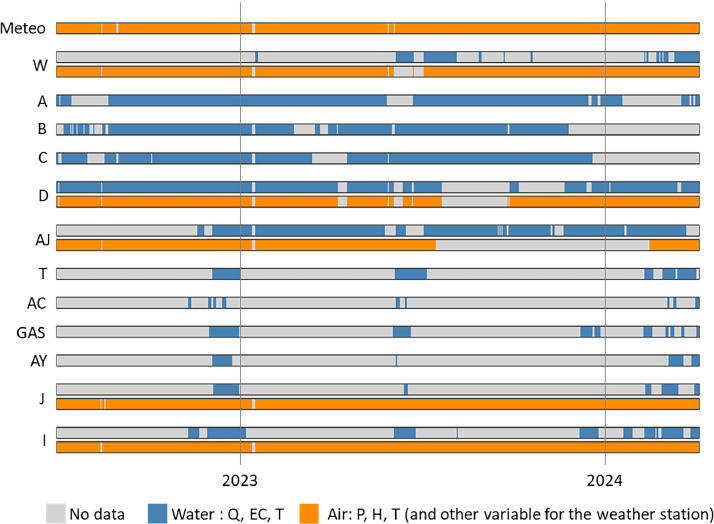
Data availability chart (representation at a daily time step).

Data consist in electrical conductivity, temperature and discharge of the 12 most frequently observed flows (A, B, C, D, W, AJ, T, GAS, AC, AY, I, and J; location Fig. 1b, Table 1); air temperature, pressure and humidity inside the galleries near flows D, W, AJ, J and I; and barometric pressure, solar radiation, humidity, temperature, wind speed, wind direction and pluviometry at the surface above the galleries (meteo station Fig. 1b). Data are available at an hourly time step from July 2022 to April 2024 and will be updated as stations continues to monitor. As most of these 12 flows are temporary, measurements are not available for the whole period (Fig. 2). There are also some data gaps for permanent flows (A–D), which correspond to temporary technical problems.

**Table 1:** Latitude, longitude and depth of the monitoring station inside and above LSBB’s galleries.

Station	Latitude	Longitude	Depth from surface (m)
D	43°56′11.70"N	5°27′57.79"E	33.3
C	43°56′22.69"N	5°28′22.67"E	256
B	43°56′27.71"N	5°28′34.11"E	421.5
A	43°56′28.94"N	5°28′36.93"E	442.2
W	43°56′32.00"N	5°28′52.69"E	437.4
AJ	43°56′13.97"N	5°29′3.46"E	359
T	43°56′1.00"N	5°29′4.08"E	195.7
AC	43°56′0.62"N	5°29′1.31"E	183.5
GAS	43°56′1.33"N	5°29′0.93"E	191.7
AY	43°55′51.92"N	5°29′3.10"E	104.1
I	43°55′46.85"N	5°29′5.63"E	60.2
J	43°55′46.83"N	5°29′4.27"E	58.2
Meteo	43°56′10.84"N	5°27′56.53"E	0

### 3.3 Location and Structure of the Data

The data can be accessed directly by clicking on the following links, which takes the reader to a web page containing the title, dataset description, acknowledgements, funding and contributors:

• Groundwater data from LSBB’s flows [1]:
  https://doi.org/10.26169/hplus.LSBB_water_high-frequency_monitoring
• Weather data from the surface above the galleries and from the air inside the galleries [2]:
  https://doi.org/10.26169/hplus.LSBB_air_weather_high-frequency_monitoring

These web pages also contain a box called “Download links” which allows to download various files containing the data in .zip format. Each of the fourth .zip files contains a PDF file with the conditions of use of the data and a .csv file with the data. Table 2 summarises in which .csv file the different parameters are stored, and where the .csv files can be downloaded.

**Table 2:** Location of the .csv files containing the parameters.

Type of Data and link	File name	Parameter names	Unit
*Weather data from the surface above the galleries and from the air inside the galleries:* https://doi.org/10.26169/hplus.LSBB_air_weather_high-frequency_monitoring	*LSBB_air_probe_2022-2024.csv*	*mean air temperature*	*°C*
		*mean barometric pressure*	*mbar*
		*mean humidity*	*%*
	*LSBB_weather_data_buiss_2022-2024.csv*	*global solar radiation*	*W/m²*
		*mean barometric pressure*	*mbar*
		*mean humidity*	*%*
		*mean shelter temperature*	*°C*
		*mean wind speed*	*m/s*
		*pluviometry*	*mm*
*Groundwater data from LSBB’s flows:* *https://doi.org/10.26169/hplus.LSBB_water_high-frequency_monitoring*	*Lsbb_ecoulements_tunnel_2022-2024.csv*	*flowrate*	*m3/s*
	*LSBB_multiparameter_probe_station_2022-2024.csv*	*conductivity*	*µS/cm*
		*temperature*	*°C*

The files are organized to be easily managed with programming tools such as Python and R studio as the parameters are listed in a unique column called “Name of Parameter”. The 4 files contain the following columns:

- Name of the site associated to the station: it is always “lsbb” to refer to the Low-Noise Underground laboratory, the study site;

- Name of station: it indicates the flow name (e.g. AJ, B, D) or the meteorological station called Meteo;

- Date and time of measurement: it is indicated in UTC;

- Name of parameter: as listed in the column “Parameters name” in Table 2;

- Value;

- Unit;

- Name of measuring device;

- Type of measuring device;

- E-mail of suppliers.

### 3.4 Examples of Data Representation

**Fig. 3:**
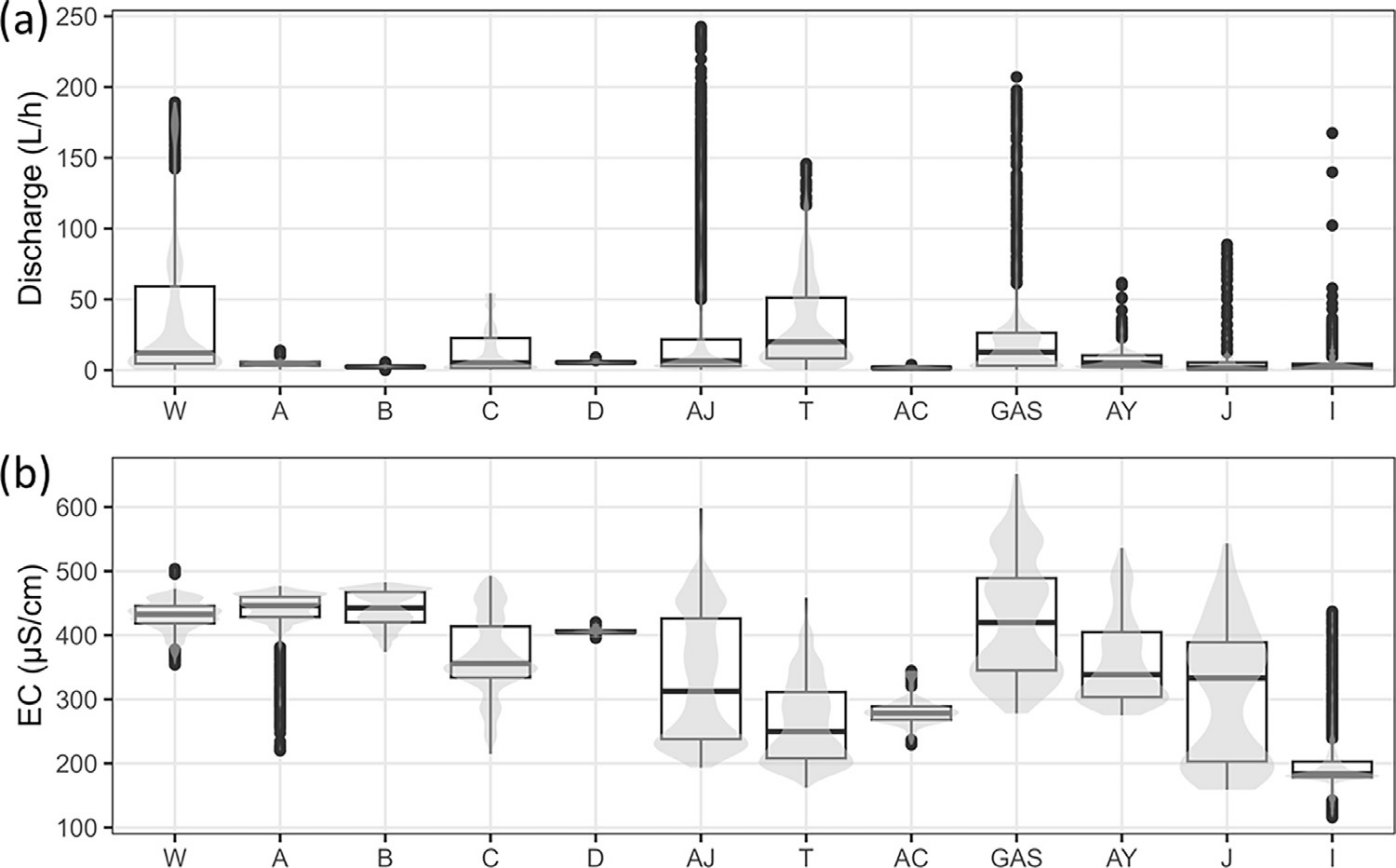
Examples of data displays: Box plots of (a) discharge and (b) electrical conductivity of the 12 unsaturated zone flows of the Fontaine de Vaucluse karst system.

**Fig. 4:**
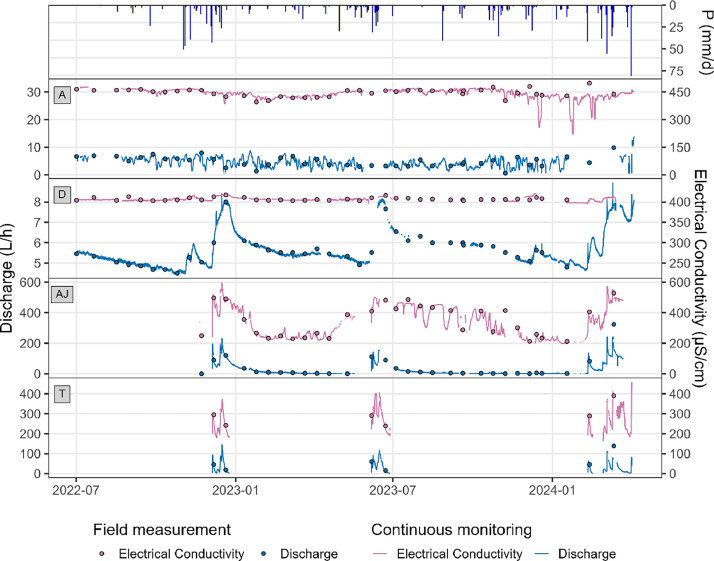
Representation of discharge and electrical conductivity of flows A, D, AJ and T with precipitations from the weather station.

These data can be synthesized by box plot to assess the variability of unsaturated zone flow parameters (Fig. 3). They can also be combined with other variables on chronicles, as shown in Fig. 4.

## 4 Experimental Design, Materials and Methods

### 4.1 Material Description

**Fig. 5:**
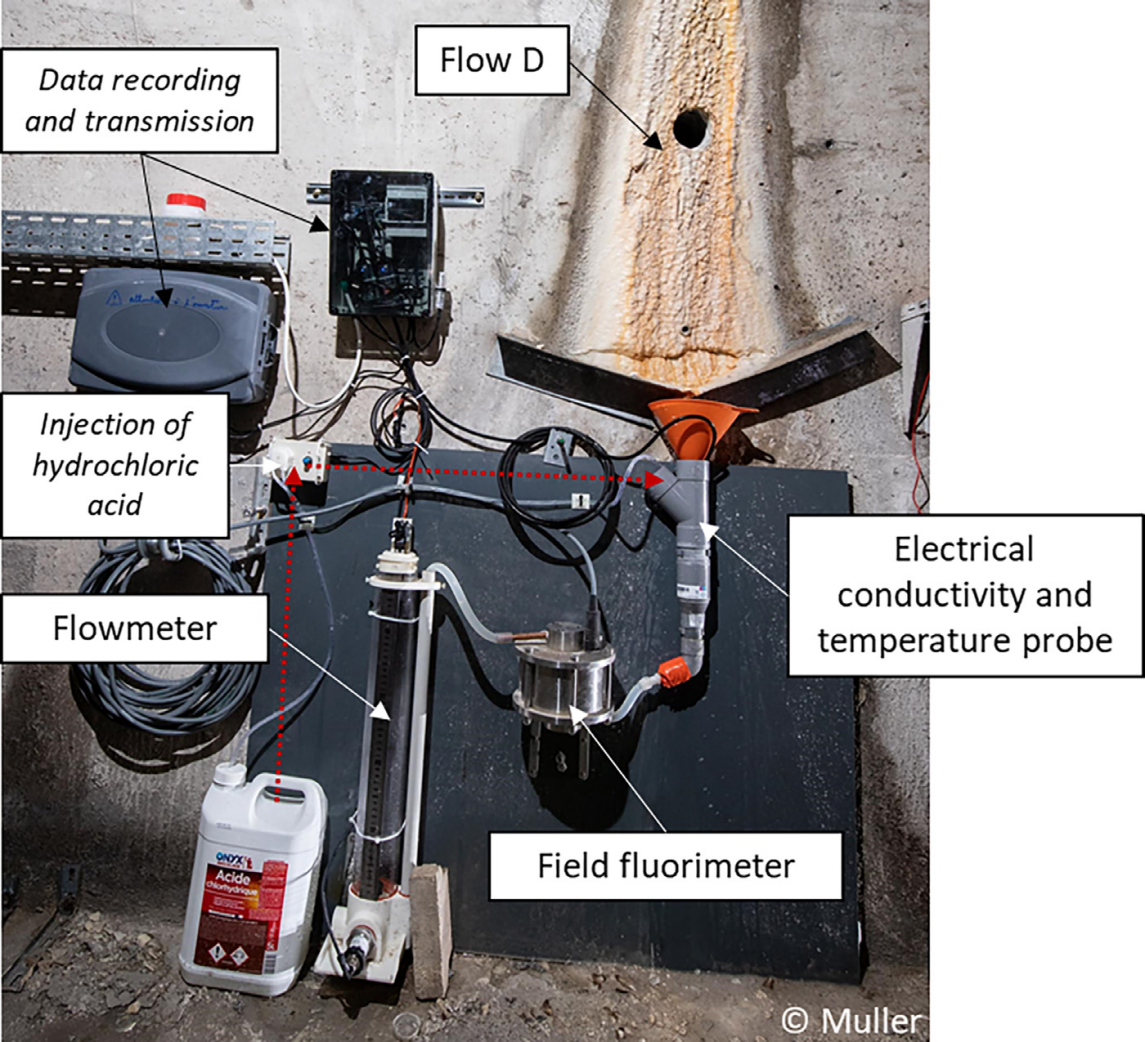
Monitoring station at flow D. Same stations are present at flows A, B and C; and same station without the field fluorimeter is installed at flows W, AJ, T, GAS, AC, AY, I, J. Fluorimeter data are not considered in this paper.

The 12 continuous monitoring stations installed at flows A–D, W, AJ, T, GAS, AC, AY, I and J are built in the same model (Fig. 5).

The water that flows along the wall is collected by metal bars towards the funnel conducting water in a PVC pipe. A probe measuring electrical conductivity and water temperature is inside the PVC pipe below the funnel. The pipe shrinkage bellows the funnel supports the probe and allows for the water storage necessary for measurement. Thanks to the pipes, water is transported either towards a fluorimeter (at flows A, B, C and D, data not considered in this paper) or directly to the flowmeter which consists of a plexiglass tube with a pressure sensor at the bottom. When the water within flowmeter reaches a defined level, a sensor commands the opening at the bottom of the tube and close the opening when the tube is empty.

LSBB galleries are also equipped with sensors measuring air pressure, air temperature and humidity at points W, D, AJ, J and I (see location in Fig. 1). At the surface above flow D, a weather station monitors air pressure, air temperature, pluviometry, solar radiation, wind speed and wind direction.

Measurements of all parameters are taken with a time step of 1 s and instrument type is provided in Table 3. Operator intervention for sampling at a weekly to monthly interval requires the disassembling of the station for easy water sampling. During each intervention, operator also injects Hydrochloric acid (around 100 mL) to keep the station clean from calcite precipitation.

**Table 3:** Description of the measuring tools.

Measuring tool	Fabricant	Sensor reference	Parameters
Weather station	Gill Instruments Limited Saltmarsh Park, 67 Gosport Street, Lymington, Hampshire, SO419EG, UK	MetPak Pro with Integrated WindSonic	Air pressure (mbar), air temperature (°C), pluviometry (mm), wind speed (m.s^−1^) and wind direction (°), solar radiation (W.m^−2^)
Flowmeter	SICK AG Sensor Intelligence, 21, boulevard de Beaubourg, ZI Paris Est - BP 42, 77184 Émerainville	PFT-SRBX10SG1SSAAMSSZ	Pressure (mA)
Water probe	AQUALABO, 90 Rue du Professeur Paul Milliez, 94506 Champigny-sur-Marne	Sensor C4E	Electrical conductivity (µS/cm) and water temperature (°C)
Air probe	Yoctopuce Sarl, 33, route de Cartigny, 1236 Cartigny, SUISSE	Yocto-Meteo	Air pressure (mbar), air temperature (°C) and humidity (%)

### 4.2 Discharge Calculation

The flowmeter consists of a plexiglass tube in which the water is collected. It has a pressure sensor at the bottom that measures the pressure inside the tube. When the water level reaches a defined level, the draining system commands the opening of the plexiglass tube at its base to drain the water. When the tube is empty, it automatically closes to allow for refilling. In the raw data, the flowmeter provides two kind of information at 1 s intervals: the pressure inside the plexiglass tube (value in mA) and whether the tube is open (value 0 or 1).

To convert these variables into discharge data, we first estimated the volume of water inside the plexiglass tube using a linear model (ax+b) between volume and pressure. This linear model was obtained by direct experimentation on the stations within the LSBB galleries, by adding known and increasingly larger volumes (from 200 to 500 mL) of water in the plexiglass tubes (3 times at least for each flowmeter) while retrieving the corresponding pressure value in mA given by sensors (r² = 09968 to 1). The data are then averaged to 10 s time step to remove the noise associated with measurements uncertainties.

**Fig. 6:**
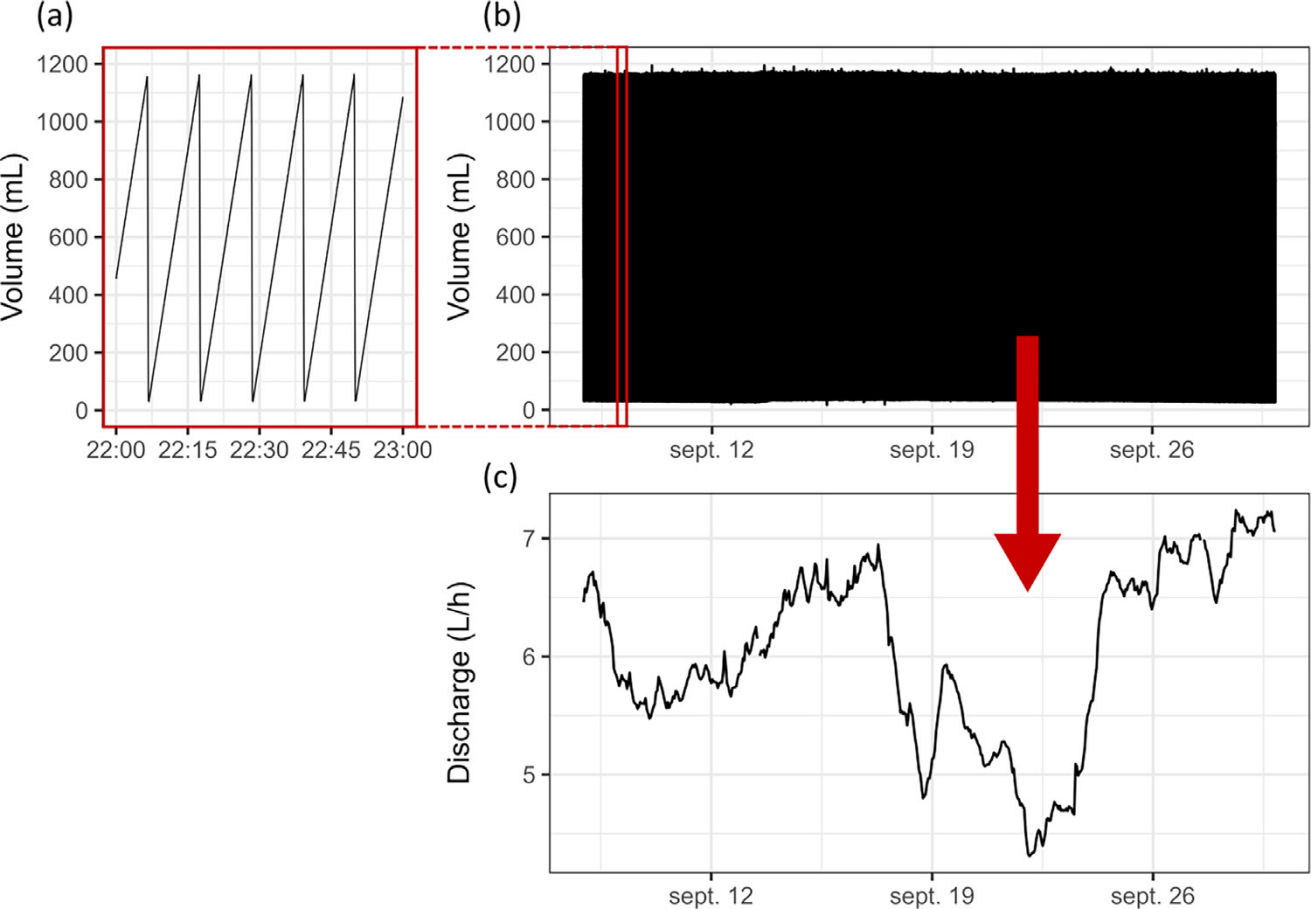
Transformation of raw data from the flowmeter (a and b) to discharge (c). Data is from flow A, September 2022.

The time series representing the volume over time shows the peaks and lows caused by the opening and closing of the plexiglass tube (Fig. 6a and b). The discharge is obtained by (i) first identifying all the extreme values of tube filling/emptying, resulting in two values (min, max) for each cycle (the lows corresponding to the plexiglass tube that has been emptied and the highs to the last values before emptying); (ii) then calculating the derivative of volume with respect to time for each cycle, which is deemed appropriate as we aim for a final hourly time step and the cycles are always shorter than 30 min. The occasional consecutive values on the same extreme (min or max) are removed as they are artefacts of sampling uncertainty. The resulting discharge value is assigned to the median time between the highest and lowest extremes (Fig. 3c). Finally, the discharge is converted to m^3^.s^−1^.

### 4.3 Data Processing and Validation

Using R software, the data have been set to an hourly time step as it is more suitable for pre-processing discharge data because of the closing and opening delay of the plexiglass tube. Data validation is performed by the following steps:

(i) Removal of erroneous values, i.e. extremes that are the result of a probe dysfunction or an operator intervention, e.g. for discharge it consists in removing values lower than 0.05 L.h^−1^ and greater than 500 L.h^−1^; for electrical conductivity lower than 100 µS.cm^−1^ and greater than 1200 µS.cm^−1^. For the precipitation, any negative values were set to 0.
(ii) Removal of isolated outliers on the discharge time series using a Hampel filter, which identifies outliers based on deviation from the median within a sliding window [9].
(iii) Control of the accuracy of the continuous measured values with respect to the point-in-time monitoring (measured at least once a month with calibrated instruments). This helps to identify potential sensor measurement drift and enables appropriate correction when necessary.
(iv) Removal of more subtle outliers on the discharge, electrical conductivity and temperature time series caused by the regular operator intervention for sampling water and cleaning the monitoring station. Those can be easily identified since the date and time of each intervention are well-documented.
(v) Removal of values when the flow is inactive (only A, B, C and D are permanent, the other 8 are temporary). For this purpose, both electrical conductivity and water temperature values are set to Non Available (NA) when the discharge is zero.

## Limitations

The first limitation to mention is that for temporary flows (all flows except A, B, C and D), data gaps due to temporary technical problems can be interpreted as flow inactivity. However, flow inactivity is less random than technical problems as it is related to environmental conditions such as rain.

Second, discharge is used as an indicator of inactive flow in order to remove electrical conductivity and temperature data while the flow is dry. However, some problems with opening or closing the Plexiglas tube of the flowmeter (e.g. calcite buildup) may lead to the misinterpretation of the flow activity, and therefore to the deletion of accurate temperature and electrical conductivity data.

## Ethics Statement

Authors have read and follow the ethical requirements for publication in Data in Brief. This work meets these requirements and authors confirm that the current work does not involve human subjects, animal experiments, or any data collected from social media platforms.

## CRediT authorship contribution statement

**Leïla Serène:** Writing – original draft, Conceptualization. **Guillaume Cinkus:** Writing – review & editing, Visualization, Formal analysis, Conceptualization. **Naomi Mazzilli:** Writing – review & editing, Supervision, Methodology, Conceptualization. **Jean-Baptiste Decitre:** Methodology. **Christophe Emblanch:** Conceptualization, Methodology. **Franck Tison:** Methodology. **Julien Dupont:** Methodology. **Milanka Babic:** Methodology. **Roland Simler:** Methodology. **Matthieu Blanc:** Methodology.

## Data Availability

OREMEHigh-frequency monitoring of discharge, electrical conductivity and temperature of flows from the low noise underground laboratory (LSBB – unsaturated zone of the Fontaine-de-Vaucluse karst system) (Original data). OREMEAmbiant air data inside and atmospheric data outside LSBB artificial gallery (unsaturated zone of the Fontaine-de-Vaucluse karst system) (Original data).
